# Peptide-Based PROTACs: Transitioning from Static Paradigm to a Dynamic Landscape within Targeted Protein Degradation

**DOI:** 10.1021/acs.bioconjchem.6c00029

**Published:** 2026-03-30

**Authors:** Xinchen Lu, Quanyin Hu

**Affiliations:** † Pharmaceutical Sciences Division, School of Pharmacy, University of Wisconsin−Madison, Madison, Wisconsin 53703, United States; ‡ Carbone Cancer Center, School of Medicine and Public Health, University of Wisconsin−Madison, Madison, Wisconsin 53703, United States; § Wisconsin Center for NanoBioSystems, School of Pharmacy, University of Wisconsin−Madison, Madison, Wisconsin 53703, United States

## Abstract

Proteolysis-targeting chimeras (PROTACs) are becoming a powerful therapeutic strategy, enabling event-driven elimination of disease-causing proteins. While small-molecule PROTACs have advanced rapidly, their broader application is constrained by limited target scope, dependence on druggable binding pockets, and susceptibility to the hook effect. Peptide-based PROTACs (pPROTACs) have gained attention as a complementary platform, leveraging the specificity, modularity, and structural adaptability of conjugates to expand the range of degradable targets, including proteins traditionally considered undruggable. This review summarizes recent advances in classical pPROTAC design and conjugation strategies and highlights emerging nonclassic and deconstructive pPROTAC architectures that reassemble functional modules through supramolecular or genetic approaches. Further, bioPROTAC, as a protein-based degrader enabled by genetic encodability, is discussed. Together, these strategies position pPROTACs as an evolving class of targeted protein degradation agents that complement small-molecule approaches.

## Graphical abstract

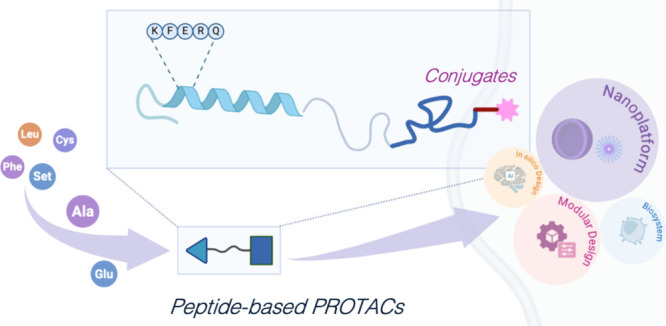

## 1 Introduction

By enabling catalytic degradation of disease-causing proteins rather than occupation-driven inhibition, proteolysis targeting chimeras (PROTACs) have expanded the therapeutic and conceptual view of pharmaceutical science.^1^ The concept of PROTACs is inherently inclusive. Under the current research context, any molecular or supramolecular construct capable of catalyzing target protein degradation can fall under this scope. Current mainstream modalities mainly include small-molecule-based, peptide-based, antibody-based, nanoparticle-based, “hybrid” degraders, and so on.^2–5^ With the innovation of their basic constructs and crosstalk with biology and materials science, the list is continuously expanding.

Peptide-based PROTACs (pPROTACs) have the longest history among all modalities, since the foundational studies that first defined the PROTAC concept were originally built upon pPROTACs, back in 2001 ^6^ (Figure 1). Sakamoto et al. chimerized small molecules with a phosphopeptide moiety that recruits β-TRCP, thereby successfully recruiting MetAP-2 to SCF^β‑TRCP^ for ubiquitination. The PROTAC concept had not yet evolved into a mature therapeutic modality as it has now, due to limited membrane permeability and subsequently unsatisfactory cellular protein degradation arising from a highly charged E3 recruitment peptide moiety. The technology re-entered the spotlight around 2015, largely driven by the rapid rise of small-molecule PROTAC research. The identification of small-molecule ligands capable of recruiting E3 ubiquitin ligases, particularly von Hippel–Lindau tumor suppressor protein (VHL) and cereblon (CRBN), enduing this bifunctional molecule a lighter molecular weight, and thus advantageous on druggability.^7^ Currently, PROTACs have entered the clinical stage and center on cancer therapy, with multiple candidates such as ARV-110 and ARV-471 in phase I/II trials. Besides tumors, targeted protein degradation (TPD) drugs aiming at autoimmune diseases and neurodegenerative diseases are expected to obtain early stage clinical results.^8,9^

**Figure 1:**
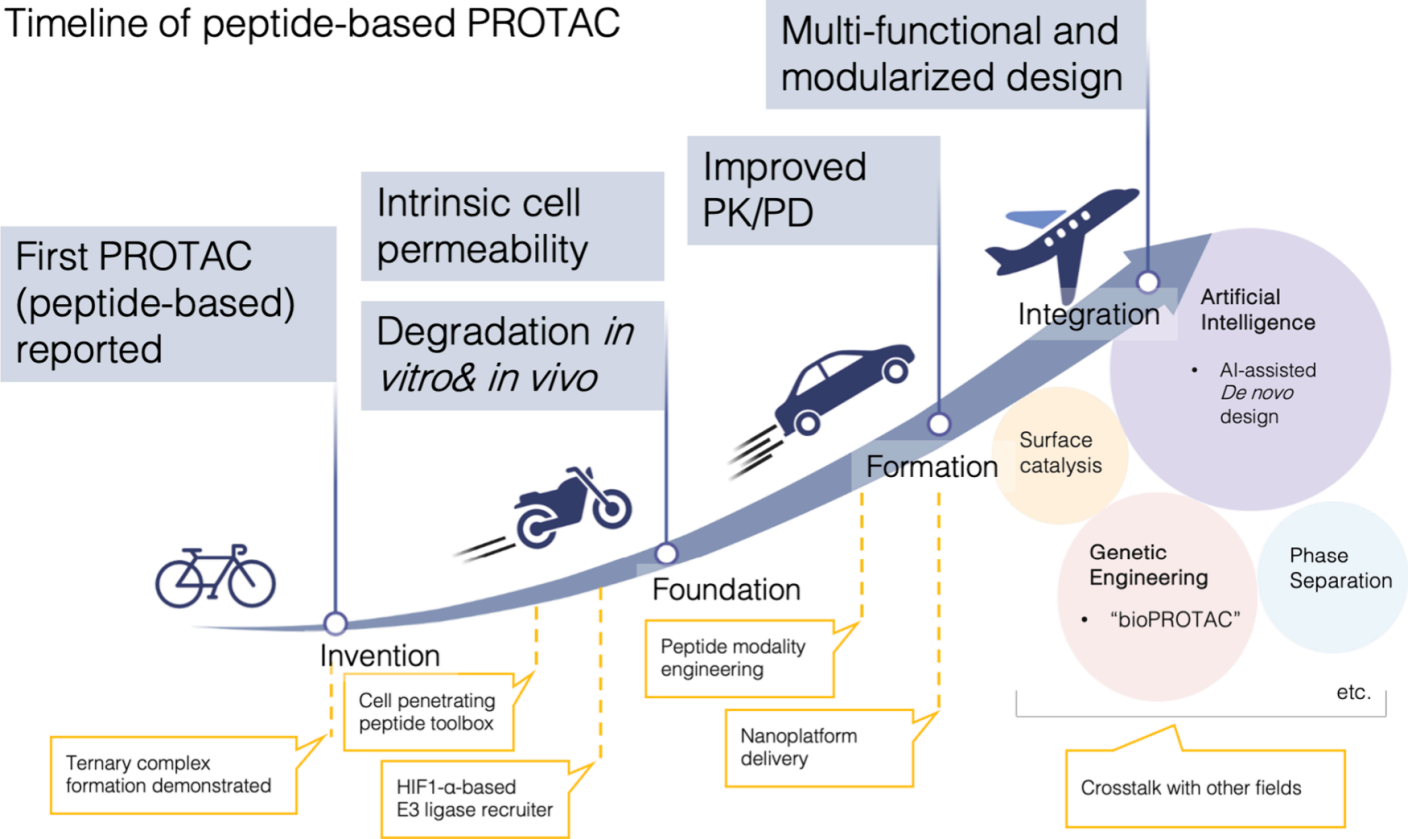
Timeline and conceptual evolution of pPROTACs. The progressive development of pPROTACs from early proof-of-concept studies to increasingly sophisticated and modularized therapeutic strategies is illustrated. Initial efforts demonstrated ternary complex formation and peptide-based PROTAC feasibility, followed by foundational advances in peptide modality engineering and CPP-based intracellular delivery. Subsequent stages emphasized improvements in intrinsic cell permeability, target degradation efficacy both in vitro and in vivo, and pharmacokinetic and pharmacodynamic (PK/PD) properties, often supported by nanoplatform-assisted delivery. Most recently, pPROTAC design has evolved toward multifunctional and modular architectures, integrating surface catalysis, genetic engineering-derived bioPROTACs, artificial intelligence (AI)-assisted de novo design, and phase separation. These advances highlight the expanding design space, cross-disciplinary integration, and translational potential of the pPROTACs. Created in BioRender. LU, K. (2026) https://BioRender.com/1b9m6ht

Even so, these developments do not imply the demise of the pPROTACs. On the contrary, the field is continuously addressing the key challenges. Regarding market analyses, the global peptide therapeutics market is growing strongly, with peptides representing 8% of drugs approved by the FDA over the past decade.^10^ High specificity and favorable safety profiles make peptide drugs attractive in certain circumstances; meanwhile, technological advances in stability and cell penetration have largely expanded the range of drug-like peptide candidates.^11^ In the case of pPROTACs, structural optimization and crosstalk with technologies from other fields have jointly propelled their rapid development (Figure 1). First, efficient peptide-based recruiters of various E3 ligases have been identified, including VHL, SCF^β–TRCP^, and KEAP1.^6,12,13^ The heptapeptidic HIF1α-derived sequence was utilized as the minimum recognition domain for VHL.^14,15^ Its relatively small size and high affinity to VHL have made it a common choice of pPROTAC design at the early stage.

To address poor permeability, cell-penetrating peptides (CPPs) hold great importance as tools for pPROTAC modification.^16^ CPPs rapidly internalize cargoes across the cell membrane, which are generally categorized into cationic type that are rich in basic residues, amphipathic type that contains both hydrophobic and hydrophilic regions, and hydrophobic type that interacts mainly with lipid bilayers.^17,18^ More than 25 CPP-relevant therapies have been under clinical evaluation, indicating the significant translational value of this strategy.^19^ The expansion of the CPP toolbox has led to more streamlined and effective pPROTAC design, along with efficient E3-binding sequences, altogether forming an established “formula” for the rational design of pPROTAC. Meanwhile, the evaluation of efficacy progressed from in vitro assays to in vivo investigations, with proof of in vivo degradation and therapeutic effects being reported.^15,20,21^ An ERα-targeting pPROTAC with an N-terminal aspartic acid cross-linked stabilized peptide ERα modulator as binder to protein of interest (POI) inhibits breast tumor growth in the MCF-7 xenograft mouse model without body weight change.^20^ Cell cycle-related and expression-elevated protein in the tumor (CREPT) was identified as a novel cancer target and degraded by a cell-permeable pPROTAC to treat pancreatic cancer in vivo.^21^ Consequently, pPROTACs achieved robust membrane permeability, allowing effective target degradation both at the cellular level and in vivo at this stage (Figure 1).

In parallel, peptide modality engineering technologies have largely improved the stability and potency of pPROTACs in a wide range of studies^20,22–24^ (Figure 1). By engineering secondary structure of linear peptides, molecular conformations would be constrained, leading to structural stability and potentially better efficacy and better druggability as well. Technologies such as stapling and cyclization preorganize bioactive structures, and have inspired multiple clinical programs.^25^ Over 16 cyclic peptide drugs have been approved globally, with sustained translation since 2000.^26^ ALRN-6924, being the furthest developed among stapled peptides, entered phase I/Ib trials in acute myeloid leukemia, myelodysplastic syndromes, breast cancer and solid tumors.^27^ Beyond conformational constraints, amino acid (AA) substitution represents a foundational strategy. The incorporation of D-amino acids and other noncanonical residues has emerged as a powerful approach to bypass enzyme recognition and improve permeability, while preserving functionality.^28,29^

At the next stage, efforts regarding formulation and delivery strategies have been incorporated into mainstream pPROTAC research (Figure 1). The leverage of nanoplatforms has proved highly effective in delivering sensitive biologics, including peptides.^30^ Through the rational design of delivery platforms, including nanoparticles, liposomes, polymeric carriers, and biomimetic systems, the “last mile” to peptide translation could be systematically addressed. Nanotechnologies provide protection for cargoes in the enzymatic environment in vivo, facilitate intracellular drug release, and achieve precise site-specific biodistribution with reduced toxicity.^31^ Owing to the highly engineerable nature of peptides, besides simply loading pPROTACs on carriers, direct conjugation with biocompatible functionalized nanomaterials further extends their therapeutic possibility. Nanoplatforms not only enable efficient delivery of pPROTACs but also impart intelligence to the therapeutic system through the incorporation of functional modules. For example, cleavable linkers can confer tissue-selective pPROTAC activation and degradation.^32^ Moreover, nanoplatforms place degraders within supramolecular systems, allowing individual functional units of the degrader to be decoupled rather than confined to a single molecule and subsequently reassembled with nanomaterials to enable more complex functionalities. Collectively, nanotechnologies are bringing revolution to the field of targeting chimeras from the perspectives of both delivery and concept.^33^

Nowadays, pPROTAC is heading toward an era of integration. The conventional “formula” of these chimeras, namely, the “binder_linker_E3 recruiter, plus cell penetrating motif” mode, still continuously produces effective therapeutic entities in the upstream, namely, modality concept and drug design; meanwhile, revolutions in downstream, namely, formulation and application, are taking place by integrating advances in related fields. First, conjugates beyond CPPs are being studied to achieve a wide range of purposes, including polymeric-, liposomal-, and metallic-based conjugates, and so on. These conjugates enable pPROTACs to assemble nanoscale complexes and demonstrate sophisticated in vivo behaviors.

In addition, pPROTACs are highly modular at both the “microscopic” amino-acid level and the “macroscopic” functional-moiety level, conferring design with high flexibility. Amino acids serve as the fundamental building blocks of peptides, and extensive evidence has shown that peptide activity and selectivity can be systematically optimized through amino-acid substitution and screening strategies, a process that can be further accelerated by in silico modeling and virtual design approaches.^34^ At the macroscopic level, functional moieties can be decoupled and coupled with other modules through the formation or breakage of amide bonds, enabling flexible reconfiguration at both the design stage and the synthesis stage. This dual-scale modularity underpins the structural adaptability and rapid optimization potential of the pPROTACs.

In this review, recent advances in the design of classical pPROTACs will be highlighted along with a summary of the development and diversification of their conjugates. Nano-pPROTACs that typically involve supramolecular assembly of conjugated pPROTACs will also be included as important frontiers in pPROTAC research (Figure 2). In addition, deconstructive pPROTACs, distinguished by a ternary complex-formation–independent yet proximity-dependent degradation mechanism and characterized by the separation of POI-binding and E3-recruiting elements into distinct molecular components, will also be introduced (Figure 2). Furthermore, bioPROTACs, similarly encoded by amino acids yet characterized by increased molecular size and structural complexity compared with pPROTACs, will be reviewed, together with a perspective on current limitations and future opportunities in the field.

**Figure 2:**
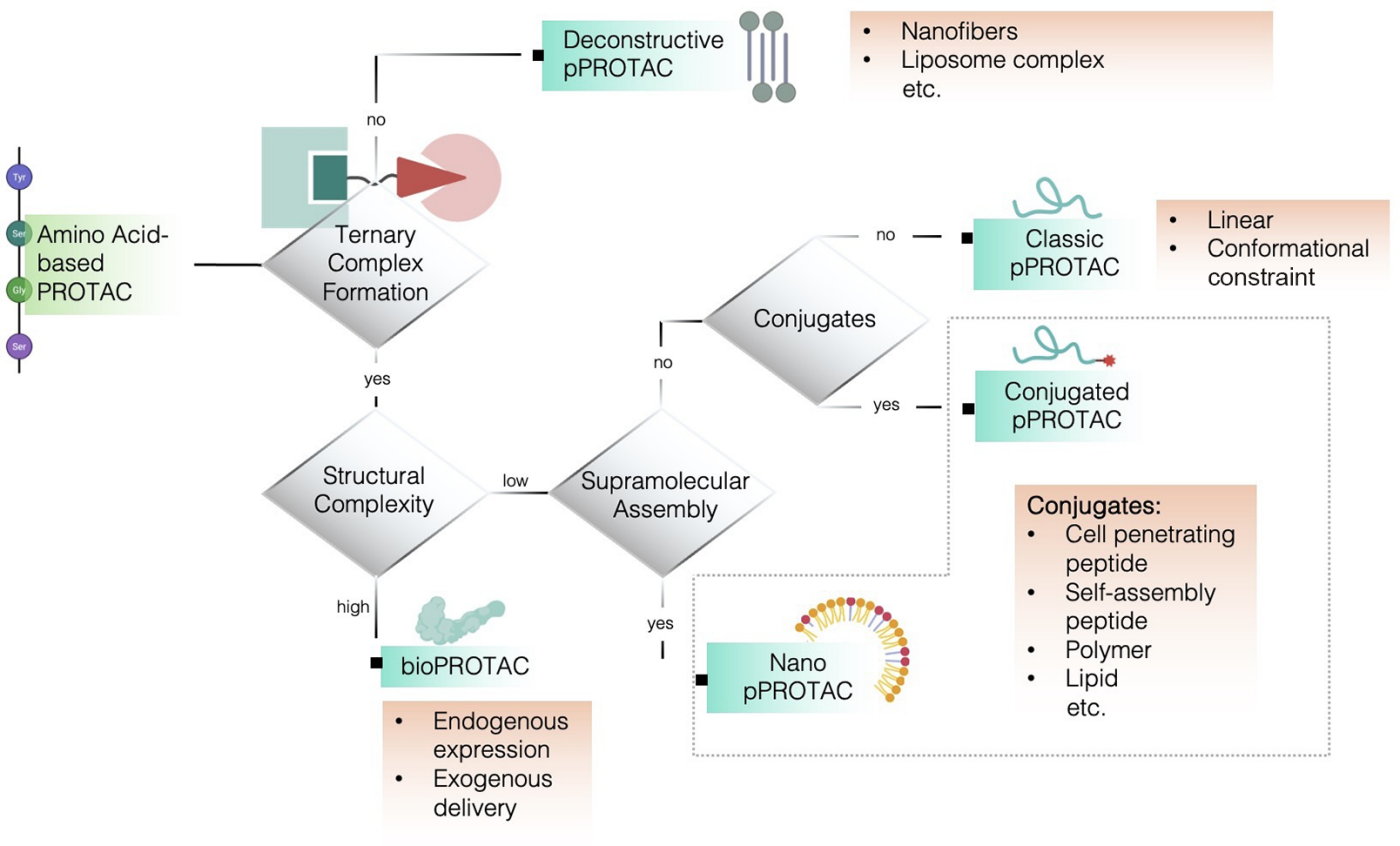
Classification framework of amino acid-based PROTACs (pPROTACs). Amino acid-based PROTACs are categorized according to their ability to form a ternary complex and their structural organization.

## 2 pPROTAC Advantage Window

While small-molecule PROTAC is a well-established framework, pPROTACs hold distinct advantages that could address some of the limitations of small-molecule ones in different scenarios (Figure 3). By leveraging the inherently specificity, modularity, and biocompatibility of peptides, pPROTACs are able to target “undruggable” proteins that are challenging for traditional small molecules and serve as an experimental platform for novel TPD modalities, owing to its high design flexibility and adaptability for conjugation with diverse materials.

**Figure 3:**
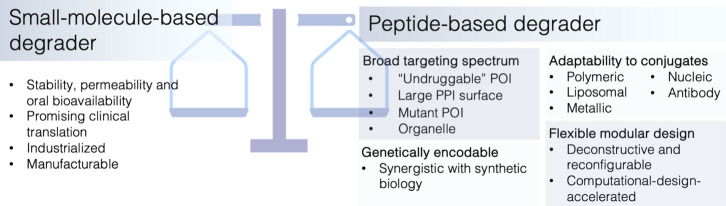
Comparison of small-molecule-based degraders and peptide-based degraders. Small-molecule degraders are characterized by favorable stability, membrane permeability, oral bioavailability, and ease of clinical translation with established industrialization and manufacturing processes. Peptide-based degraders offer broader targeting potential, including “undruggable” proteins, large or complex POIs, and are genetically encodable, allowing synthetic biology applications. Peptide-based degraders also provide flexible modular design, enabling conjugation to a variety of biocompatible materials as well as advanced design strategies such as deconstructive or computationally accelerated approaches.

Several small-molecule PROTACs have become promising drug candidates in the clinical stage.^35,36^ ARV-110 achieves meaningful prostate-specific antigen reductions and confirmed tumor responses in a subset of patients with metastatic castration-resistant prostate cancer.^35^ ARV-471 has shown robust ER degradation (>90%), favorable tolerability, and clinically meaningful antitumor activity in patients with ER+/HER2– breast cancer.^36^ Despite their rapid development and proven translational potential, their activity is often restrained by the demand for high-affinity ligands for both the POI and E3 ligases, which restricts the range of targetable proteins and the design space, particularly those with “undruggable” proteins. Although the catalyst-like action of PROTAC partly lowers the affinity requirement for the POI ligand, robust pocket occupation is still needed to form a stable ternary complex. Also, PROTACable E3 ligases are limited for small-molecule PROTACs, with CRBN and VHL being dominant choices.^1^ Given the pairing of E3 ligase for the target results in different degradation efficiencies, more E3 options are still needed.^37^ Together, these factors limit the chemical space and target spectrum, which motivates the development of alternative platforms.

Peptide drugs often combine general advantages like high specificity and potency, empowering them with a relatively wide targeting spectrum,^38^ including protein with large PPI surfaces, mutant POI, etc. “Undruggable” proteins often refers to proteins that have flat and featureless surfaces, resulting in the lack of a well-defined binding pocket, whereas peptides could have ideal pharmacological efficiency.^39^ These proteins mainly function by PPI interaction, the interaction surface of which tends to be too large to be completely blocked by small molecules. Owing to their larger molecular size, peptide drugs can engage broader PPI interfaces, representing an effective approach for targeting such proteins (Figure 3).

For instance, tumor suppressor transcription factor p53 lacks a deep enzymatic pocket, the function of which is mainly regulated through the interaction with MDM2, thus was considered undruggable traditionally. ALRN-6924, a stapled-peptide-based inhibitory agent of MDM2:p53 interaction, has advanced to clinical trials, making the protein a clinical target.^27^ Further, a triple-action pPROTAC (TAPTAC1) was reported by Bird et al., composed of BET inhibitor JQ1 and the optimized analogue of ALRN-6924. TAPTAC1 inhibited HDM2 and HDMX to maximally reactivate p53 while hijacking HDM2 to degrade oncogenic BET proteins.^40^ Tau, as an important pathological protein of Alzheimer’s disease, is challenging to regulate as a nonenzymatic protein, where pPROTAC research aims at resolving. Chu et al. designed a series of pPROTACs with Tau-targeting sequence, VHL minimum recognition domain, and a GSGS linker to engage degradation.^41^ TH006, the hit with the best potency, could decrease the Tau level in the brain of an Alzheimer’s disease mouse model. As another example, the AR DNA binding domain participates in key pathology of prostate cancer, and it also forms a dimerization interface that adopts a flat conformation, making the binding of small molecules energetically difficult. In one study, Rosetta virtual amino acid screening at key amino acid sites was performed, AR DBD-binding peptide was derived from its homodimeric structure and designed into a pPROTAC.^42^

Mutant POI has been identified as one of the dominant causes of acquired drug resistance.^43^ During treatment, target proteins can acquire mutations at key hotspot residues within small-molecule binding pockets under prolonged occupancy, undermining their efficacy. Meanwhile, peptide drugs often demonstrate a restored affinity to mutant POI (Figure 3). Rat sarcoma (RAS) mutations are important driving factors of several fatal cancers, with over 40% KRAS variant mutations in pancreatic ductal adenocarcinoma and colorectal cancer.^39^ Inhibitors targeting wild-type and multiple mutant RAS are of high therapeutic significance. Several peptide pan-RAS inhibitors were reported and showed profound antitumor activity, being a spotlight for pan-RAS inhibition.^44,45^ By re-engineering the proteomimetic pan-RAS peptide inhibitor CDH^SOS^, RAS degrader CHD^BI4^ was reported by Ongkingco et al., acting as a peptidic molecular glue through engaging RAS and MDM2 to the ternary complex.^46^ The bifunctional structure is a cross-linked helix dimer stabilized by noncovalent intramolecular interactions like knobs into hole packing, inter- and intrahelical salt bridges, and an interhelix covalent bond, inspiring a novel molecular glue platform.

Notably, organelles have been included within the scope of degradable “target” using peptides as a functional modality. The mitochondrial nanoinducers that selectively target and degrade mitochondria within autophagosomes were reported by Pan et al.^47^ The group conjugated LC3-targeting and mitochondria-targeting peptides, respectively, on gold nanoparticles through gold–sulfur reaction, creating proximity between mitochondria and autophagosomes, subsequently leading to mitochondria “degradation”. In this pattern, gold nanoparticles serve the classic “linker” role.

Another advantage of PROTAC based on AA is its adaptability to the endogenous genetic system (Figure 3). Following the central dogma of molecular biology, nucleotide sequences encode amino acids, which are subsequently assembled into peptides and proteins.^48^ AA-based PROTACs, bioPROTACs, essentially protein-like,^49^ can therefore be intrinsically integrated into the endogenous genetic system, allowing their sequences to be encoded at the DNA level and expressed intracellularly through transcription and translation, enabling crosstalk with genetic engineering technologies. Such genetic encodability could circumvent conventional challenges associated with chemical synthesis and intracellular delivery, also enabling spatiotemporal control over the PROTAC functions. Moreover, bioPROTACs are compatible with synthetic biology and protein engineering strategies, therefore allowing sophisticated functional design to fulfill precise tasks.

Collectively, these examples highlight pPROTACs as a compelling and complementary modality to small-molecule PROTACs, particularly in scenarios in which conventional chemical space and ligand availability impose intrinsic constraints (Figure 3). By exploiting the adaptable interaction surfaces of peptides, pPROTACs expand the degradable target landscape to include broader biological entities. Beyond target engagement, the modularity and genetic encodability of bioPROTACs enable the integration with protein engineering, synthetic biology, and gene delivery technologies, offering opportunities for precise control and multifunctional design. Continued advances in peptide chemistry, conjugation strategies, and nanotechnology are expected to progressively mitigate the barriers regarding peptide druggability issue. To sum up, pPROTACs represent not only an alternative degrader format, but an expanded conceptual framework for TPD, being potential in exploring previously inaccessible biology and therapeutic opportunities.

Although distinct advantages in certain scenarios are verified for pPROTACs, small-molecule PROTACs currently have a clearer laboratory-to-clinic and laboratory-to-industry translation pathway. Small-molecule PROTACs benefit from well-established medicinal chemistry optimization strategies, scalable synthetic routes, and clearer regulatory frameworks aligned with traditional small-molecule drug development. Their pharmacokinetics, manufacturability, and formulation approaches are more compatible with the existing pharmaceutical infrastructure. Additionally, small-molecule PROTACs that have already entered clinical trials can provide references for safety evaluation, dosing strategies, and translational evaluation markers. In contrast, pPROTACs are generally subjected to translational uncertainty. A “0 to 1” breakthrough is anticipated, possibly as a result of the optimized translational characteristics like stability and bioavailability, or the identification of unique biological applications that leverage their distinctive features.

## 3 Narrowly Defined pPROTACs

According to the canonical definition of PROTACs, a PROTAC is a heterobifunctional molecule composed of three essential elements: (1) a ligand that specifically recognizes the POI, (2) an E3 ligase–recruiting moiety that brings the POI into proximity with an E3 ubiquitin ligase, and (3) a linker that connects these two modules and maintains a spatial arrangement favorable for formation of the POI–PROTAC–E3 “ternary” complex, thereby facilitating ubiquitination (Figure 2).

In pPROTACs, these functional elements are primarily constructed from peptides composed of AAs linked via amide bonds. The POI-binding motif and the E3 ligase-recruiting motif are typically short peptides, generally ∼5–20 residues in length. As for the linker, pPROTACs most commonly employ PEG segments or AA-based flexible spacers, which are readily accessible through solid-phase peptide synthesis and permit the fine-tuning of ternary complex formation.

In this section, we use the term “narrowly defined pPROTACs” to refer to constructs in which the three canonical PROTAC components are covalently integrated within a single molecular entity that is capable of inducing the formation of a “ternary” POI–PROTAC–E3 ligase complex. This definition includes conjugated pPROTACs, provided that all three functional elements within the same molecule act cooperatively to mediate the formation of ternary complex formation. Conversely, we exclude multicomponent systems in which targeting and E3-recruiting elements are separate entities and rely on assembly to induce proximity.

### 3.1 Advances in Design of Classic Elements

Currently, the discovery pipeline of peptide binders for known target proteins is well established. Derivation from known protein–protein interaction (PPI) motifs and display-based library screening are dominant approaches in hit identification^50^ (Figure 4). Upon in silico docking model establishment, α-helices and β-strands from PPI hot spot sequences are preferred to be chosen as hits. Display-based library screening is a central strategy for peptide hit identification, including technologies such as phage, mRNA, and yeast display.^51–53^ They allow the rapid enrichment of high-affinity binders, including linear and cyclic peptides. In 2024, the Nobel Prize in Chemistry was awarded for AI-driven protein structure prediction and molecular design, which has highlighted the potential of computational approaches in molecular discovery. De novo peptide design has become a powerful strategy in peptide drug discovery, enabling the rational generation of functional peptide sequences guided by engineering principles rather than existing starting points found in nature.^54^ Vazquez Torres et al. leveraged deep learning to design short-peptide binders to bioactive helical peptides with picomolar affinity.^34^

**Figure 4:**
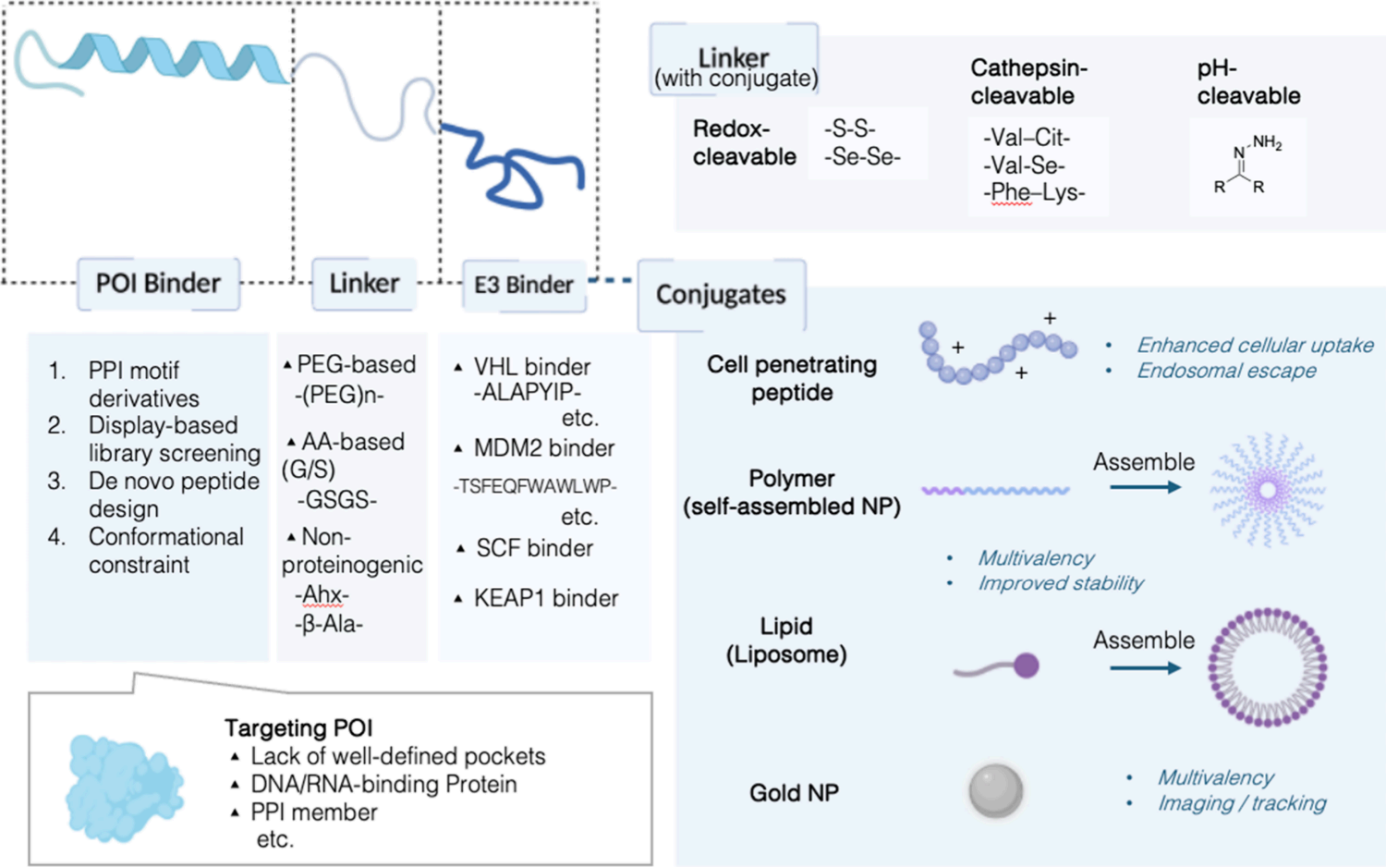
Design and conjugation strategies of the pPROTACs. pPROTACs are composed of three modular elements: a peptide binder for POI, a linker, and an E3 ligase-recruiting ligand. POI binders can be identified through PPI motif derivation, display-based library screening, or de novo peptide design, and can be modified by conformational constraint strategies, enabling targeting of diverse protein classes, including enzyme's lack of well-defined pocket, DNA/RNA-binding proteins, etc. Linkers can be engineered to incorporate cleavable motifs, such as redox-responsive, protease-cleavable, or pH-sensitive linkages, allowing conditional activation and intracellular release. The pPROTACs further enables conjugation with various functional carriers, including CPPs, self-assembled polymeric nanoparticles, liposomes, and gold nanoparticles, which enhance cellular uptake, stability, multivalency, and tracking capabilities. Created in BioRender. LU, K. (2026) https://BioRender.com/s8169vv.

Compared with a wealth of alternatives for warheads in cases of a wide range of POIs, there have been limited novel options for E3 recruiters (Figure 4). Interestingly, a hydrophobic tag, Boc3Arg, was discovered to initiate protein degradation. Instead of inducing ubiquitination, Boc3Arg ligands bind directly to the 20S proteasome and stimulate its activity.^55^ Looking ahead, the discovery of high-efficiency peptide POI binders and E3 recruiters will be greatly accelerated and optimized by AI.

Ma et al. applied ProteinMPNN and RFdiffusion to identify binding peptides for androgen receptor (AR) and VHL, followed by computational modeling with Alphafold2-multimer and ZDOCK to predict spatial interrelationships, and reported the peptide-based AR degrader MNs-Se AR PROTAC. An optimal length of 11.4 Å between the AR and VHL was obtained through analysis and achieved through a pentapeptide linker. The degrader was formulated into microneedles, and the potency of treating androgenetic alopecia was verified both in vitro and in vivo. Xu et al. utilized Rosetta to design a new peptide binder of PAK4, which was first reported in this study as a potential target for kidney cancer.^56^ Further, generated pPROTAC can serve as a post-translational knockdown tool. A p300 degrader was designed by Zhang et al. through Rosetta, in which the cysteine-histidine-rich 1 domain of p300 was targeted, demonstrating good efficacy in prostate cancer xenograft models. For novel disease targets without an existing promising ligand, AI-generated peptide sequences provide options at substantially lower time and economic costs.

De novo peptide design has equal potential in developing other TPD modalities. Similar to PROTAC components, other TPD elements with higher affinity and superior functionality can be discovered with the aid of computational design. LYTACs and PROTACs both belong to targeted protein degradation strategies. PROTACs hijack the proteasome system, and LYTACs exploit the lysosomal system; therefore, the POI binder in LYTAC design is linked to a lysosome-targeting moiety.^57^ Huang et al. developed endocytosis-triggering binding proteins and POI (including EGFR, PD-L1, HER2, etc.) binders through the Rosetta de novo binder design, creating all-amino-acid-based LYTACs.^58^ Notably, these bio-orthogonal endocytosis-triggering binding proteins bypassed competition with native ligands and requirements for chemical modification.

For pPROTACs in which the three canonical PROTAC components are integrated into a single peptide molecule, functional conjugates of pPROTACs have emerged for multiple purposes. In these systems, peptide-based PROTACs are chemically or supramolecularly coupled to auxiliary functional modules that endow additional properties while preserving the core degradation mechanism.

### 3.2 CPPs as Conjugates

One major category of pPROTAC conjugates comprises cell-penetrating ones, in which pPROTACs are linked to either classic CPPs or membrane-active motifs to facilitate intracellular delivery (Figure 4). These conjugation strategies can enhance cytosolic access of pPROTACs, thereby improving the degradation efficiency in cellular contexts. CPPs are a diverse class of short peptides capable of translocating across cellular membranes and facilitating intracellular delivery of conjugated cargos.^17^ The most widely used CPPs include cationic peptides such as HIV-1 TAT and polyarginine, which rely on high arginine content to promote membrane interaction and uptake,^59^ as well as amphipathic CPPs, which combine charged and hydrophobic residues to enhance membrane insertion.^60^ These CPPs have been extensively validated as delivery vectors for biomolecules and are commonly adopted in CPP–pPROTAC system.^15,54,61,62^ Schneekloth et al. first fused polyarginine sequences to pPROTAC to facilitate intracellular translocation,^15^ and this pattern was followed by other studies.^21,63^ Wang et al. utilized TAT as cell-penetrating conjugate and achieved FOXM1 degradation in vivo.^61^ Shi et al. developed intracellular DHHC3 degradation as a potential cancer therapy to improve immune checkpoint blocker resistance, also involving TAT as CPP conjugate for pPROTAC.^62^

### 3.3 Nano-pPROTAC

Another category involves delivery-oriented conjugates, forming nanoscale carriers based on material properties (Figure 2). Peptides usually have excellent hydrophilicity and have favorable chemical modifiability to a wide range of materials, and are therefore well suited for the modification of nanoparticles.^64^ Lipid conjugation can promote plasma protein binding and extend circulation half-life,^65^ whereas polymeric or nanocarrier-based formulations enable protection from proteolytic degradation and allow controlled release^66^ (Figure 4). While unmodified pPROTACs often suffer from rapid clearance and limited bioavailability, these approaches are particularly effective for in vivo applications. Meanwhile, functional conjugation has also been exploited to achieve spatiotemporal control over the pPROTAC activity. Examples include conjugates bearing cleavable linkers responsive to intracellular redox conditions, enzymatic activity, or pH, as well as stimulus-activated designs. In such systems, the conjugated moiety functions as a regulatory element that restricts degrader activity until exposure to specific environmental triggers.

Self-assembling pPROTACs provide a minimalist yet powerful approach for constructing nanocarriers without the need for external polymeric structures. By rationally integrating pPROTAC motifs with self-assembly sequences, these systems leverage intrinsic noncovalent interactions to spontaneously organize into supramolecular architectures.^67,68^ Importantly, self-assembled pPROTAC nanostructures can simultaneously serve as both the therapeutic agent and the delivery vehicle, thus eliminating carrier-associated complexity and reducing formulation heterogeneity. Such carrier-free designs enable a high drug loading efficiency. Overall, self-assembling pPROTACs offer a streamlined and adaptable platform for expanding the translational potential of pPROTACs

Conjugating pPROTAC to amphiphilic NFTP peptide with a cathepsin-B cleavable linker and branched with a CPP moiety, Zeng et al. developed a self-assembled peptide PROTAC prodrug system for tumor-specific POI degradation.^69^ Moon et al. utilized the self-assembly of amphiphilic peptide-derived PROTAC to realize tumor-targeted and durable programmed cell death ligand 1 (PD-L1) degradation, with FF peptide as linker.^70^ In this work, a linker composed of two phenylalanine residues was employed as a self-assembly module. Driven by its hydrophobicity, it forms nanoparticles, which promote lysosomal degradation of PD-L1 through nanoparticle-mediated sequestration, while the pPROTAC monomer simultaneously triggers target protein degradation via the canonical ubiquitin–proteasome pathway. Similarly, Jeong et al. also utilized the self-assembling peptide linker FF to produce uniform spherical nanoparticles assembled by EGFR-targeting pPROTACs. The NanoTAC system effectively eliminated both wild-type and L858R/T790 M mutant EGFR and suppressed tumor growth by 88.3% in colon and lung tumor models.^71^

By covalently linking pPROTACs to polymeric backbones, these constructs self-assemble into nanoscale carriers. Lu et al. conjugated pPROTAC and PEG2k-DSPE with disulfide bonds, forming STAT3 and beta-catenin dual degradation micelles as colorectal cancer therapy. The micelles responded to the reductive tumor microenvironment and exhibited a tumor-specific pPROTAC release.^63^

Gold nanoparticles hold strong reaction activity for sulfur-containing ligands and are widely exploited for the conjugates with peptides bearing terminal cysteine residues or thiol-functionalized linkers^72^ (Figure 4). Gold nanoclusters connecting HER2-targeting peptides and CRBN-targeting ligands for HER2 degradation were reported by Wang et al. The gold nanoclusters were synthesized by reduction of HAuCl4 with glutathione, and HER2-targeting peptides were conjugated through gold–sulfur coordination. The system improved the intracellular delivery and the stability of peptides, and also showed degradation efficiency on membrane proteins.^73^

Collectively, functional conjugates of pPROTACs represent a versatile extension of the peptide-based degradation paradigm, enabling modular optimization of delivery, stability, and controllability. As conjugation chemistries and delivery technologies continue to mature, these hybrid designs are expected to play an increasingly important role in translating pPROTACs from conceptual tools to therapeutically viable modalities.

### 3.4 Conformational Constraint

Engineering linear peptides as conformational constrained ones represents a resolution to improve pharmacological activity and stability. Conformational constraint keeps the peptide closer to its bioactive conformation, therefore having a favorable binding affinity to target the POI. Moreover, the constraint limits the exposure of cleavage sites, which leads to lower metabolic clearance and longer systemic half-life. Cyclic peptides feature the closure of AA residues at their N- and C-terminal or side chains, whereas one of the subsets, peptide stapling, focuses on stabling the α-helical conformation that is critical to bioactivity of many peptides with side chain linkage. These advances have been integrated as tools for the improvement of pPROTAC hits.

Utilizing stapling strategy to help stabilize the α-helix sequences of warheads is commonly reported in research studies.^20,22,24,74^ Stapled pPROTAC showed enhanced on-target activities compared to the linear analogue.^20^ Whittaker et al. applied a hydrocarbon-stapled peptide as the protein kinase A-targeting agent, and the StIP-TAC molecule they designed promoted protein kinase degradation in a proteasomal-dependent fashion and caused downregulation of kinase signaling.^22^ Interestingly, an AR-targeting pPROTAC study first reported the instance of hydrocarbon stapling as stabilization of the β-sheet secondary conformation.^23^ The double-stapled pPROTAC underwent conformational stabilization by stapling at both the warhead region and the MDM2 recruiter segment. Compared to its linear analogue, the double-stapled pPROTAC exhibited better permeability, with its in vivo antitumor effects verified.

## 4 Nonclassic and Deconstructive pPROTACs

Beyond the canonical architecture in which a peptide-based PROTAC integrates a POI-binding warhead, an E3 ligase recruiter, and auxiliary delivery motifs into a single molecular entity, emerging studies have begun to challenge the necessity of this monolithic design. Nonclassic and deconstructive pPROTAC strategies reimagine targeted protein degradation by functionally separating individual modules and reassembling them through genetic encoding, supramolecular assembly, or stimuli-responsive organization. These approaches preserve the central principle of proximity-induced ubiquitination while offering new solutions to the intrinsic limitations of classical pPROTACs, such as molecular size, synthetic complexity, and constrained stoichiometry. By expansion of the architectural and mechanistic design space of peptide-based degraders, deconstructive pPROTACs represent an important frontier in the evolution of targeted protein degradation technologies.

### 4.1 Modular Deconstruction of pPROTAC

pPROTACs are inherently modular in design, typically comprising a target-binding warhead, an E3 ligase ligand, a linker that spatially organizes these two components, and, in the majority of cases, an additional cell-penetrating motif. Through the coordinated functions of these modules, pPROTACs induce proximity between the POI and recruited E3 ligase, thereby triggering POI ubiquitination and subsequent proteasomal degradation. However, these functional modules are not strictly required to copresent within a single molecular entity. A series of creative studies have deconstructed the conventional pPROTAC architecture, physically separating individual modules and reassembling them through alternative strategies such as self-assembly or supramolecular organization, while preserving proximity induction of POI and E3. The deconstructive design not only expands the architectural design of pPROTACs but also offers potential solutions to the intrinsic limitations of classical designs, including molecular size, synthetic complexity, and suboptimal pharmacokinetic or delivery properties. Notably, in cases of deconstructive pPROTAC, the stoichiometric ratio between the POI binder and the E3 ligase binder is not necessarily 1:1, allowing for more optimized ratios for improved degradation efficiency to be employed.

Zhang et al. reported an intracellular fabricated Nano-PROTAC system, where two precursors consisting of hydrophilic assembly peptides and POI/E3 binders conjugate through click reaction into a POI degradation nanostructure under high GSH levels in cancer cells^75,76^ (Figure 5). Specifically, the peptide GNNQQNY that can form amyloid fibrils is used as a self-assembly motif, branched with alkyne or azide groups as linkers between nanofibers. GHK-stabilized Cu^II^ can be reduced to Cu^I^ by high GSH levels and catalyzes click chemistry, inducing assembly of the two precursors. As a result, POI binders and E3 binders were distributed around the amyloid fibrils formed, recruiting multiple POI and E3 ligase molecules to participate in the ubiquitination process. Notably, this mode eliminates the impact of the “hook effect” on the PROTAC-mediated degradation. Conventional PROTACs often exhibit a decline in degradation efficiency at high concentrations, namely the hook effect, in which preferential formation of binary complexes with either the POI or the E3 ligase competes with ternary complex assembly, thereby limiting their efficacy at high doses.^77^

**Figure 5:**
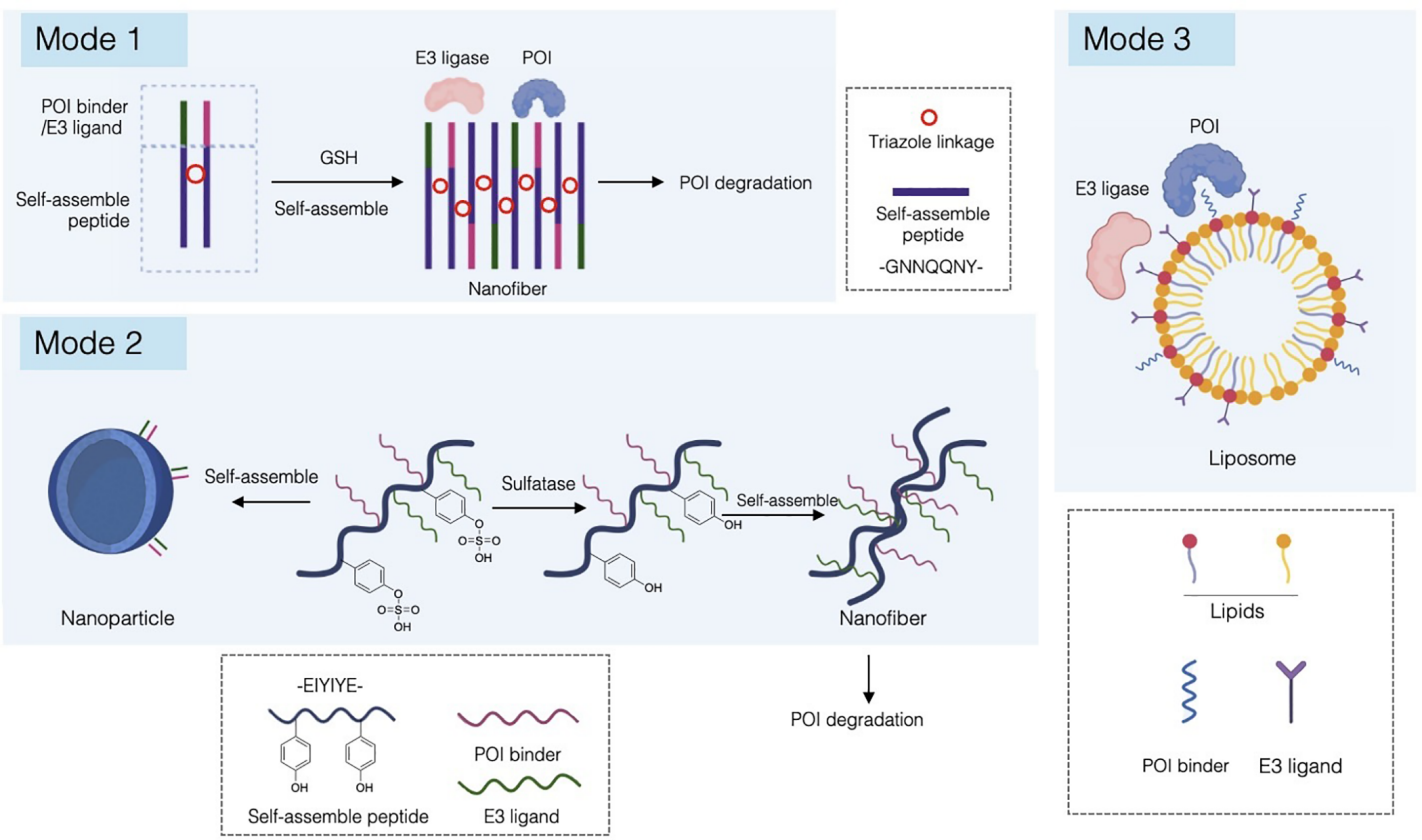
Deconstructive and supramolecular assembly strategies for pPROTACs. Schematic illustration of modularly deconstructed pPROTAC architectures in which the POI-binding and E3 ligase-recruiting modules are physically separated and reassembled through self-assembly or supramolecular organization to induce proximity-driven protein degradation. Mode 1: Intracellular fabrication of Nano-PROTACs was via redox-triggered click chemistry. Mode 2: Enzyme-responsive supramolecular PROTAC assembly. Mode 3: Liposome-based deconstructive pPROTACs. Created in BioRender. LU, K. (2026) https://BioRender.com/s8169vv.

Stimuli-responsive assembly of POI- and E3-ligase binders into supramolecule architecture can lead to stimuli-specific protein degradation, enhanced accumulation, and retention. Chen et al. utilized the enzyme-responsive structure switch of a sulfated peptide and designed a strategy for in situ formulation of antineoplastic supramolecular PROTACs, where coassembling of the sulfated peptide with VHL ligand and Bcl-xL ligand was involved (Figure 5). The pro-Supra-PROTAC showed an efficient internalization and underwent enzyme-responsive assembly into Supra-PROTACs with a nanofibrous morphology merely in cancer cells overexpressing sulfatase.^78^

Identifying Prostaglandin E Synthase 3 (PTGES3) as a potential hepatocellular carcinoma target, Liu et al. conjugated the lipid (DSPE-PEG2000-MAL) with the PTGES3-binding peptide and CRBN ligand pomalidomide, respectively, and formulated a liposome complex (Figure 5). The degradation efficiency of the POI by the liposome can be adjusted by altering the ratio of lipids conjugated with the PTGES3-binding peptide and pomalidomide.^79^

### 4.2 bioPROTACs

Peptides and proteins share a common chemical foundation, both being composed of AAs linked by amide bonds and are traditionally distinguished by size, structural complexity, and functional organization. Peptides are generally shorter and often lack a stable tertiary structure, whereas proteins are larger polypeptides that typically adopt well-defined 3D folds and functional domains. BioPROTACs refer to AA-based PROTACs that are protein-like functional biomacromolecules. These bioPROTACs can be broadly divided into two modes: endogenous expression via genetic engineering and exogenous delivery following expression in nonmammalian systems. bioPROTACs maintain the core principle of POI ubiquitination and work both as biological tools for target protein knockdown and as a novel therapeutic modality. They have been proven efficient in targeting undruggable targets, especially DNA/RNA-binding proteins that lack a potent small molecule inhibitor. Moreover, for proteins localized to subcellular compartments where endogenous E3 ligase accessibility is limited, bioPROTACs eliminate the dependence on E3 accessibility, and therefore has the potential to complement conventional PROTACs in targeting otherwise intractable proteins.

Lim et al. engineered fusion proteins that consist of a target binding domain and an E3 ligase as biodegraders.^49^ By introducing plasmids encoding bioPROTACs, intracellular expression of the degraders was achieved, enabling the recruitment of multiple E3 ligases, including SPOP. Notably, eight out of ten tested E3 ligases were capable of mediating efficient degradation, culminating in successful elimination of the protein–protein interaction target PCNA (Figure 6). This study highlights the flexibility of the bioPROTAC design and demonstrates that a broad range of E3 ligases can be harnessed to degrade challenging PPI-driven targets.

**Figure 6:**
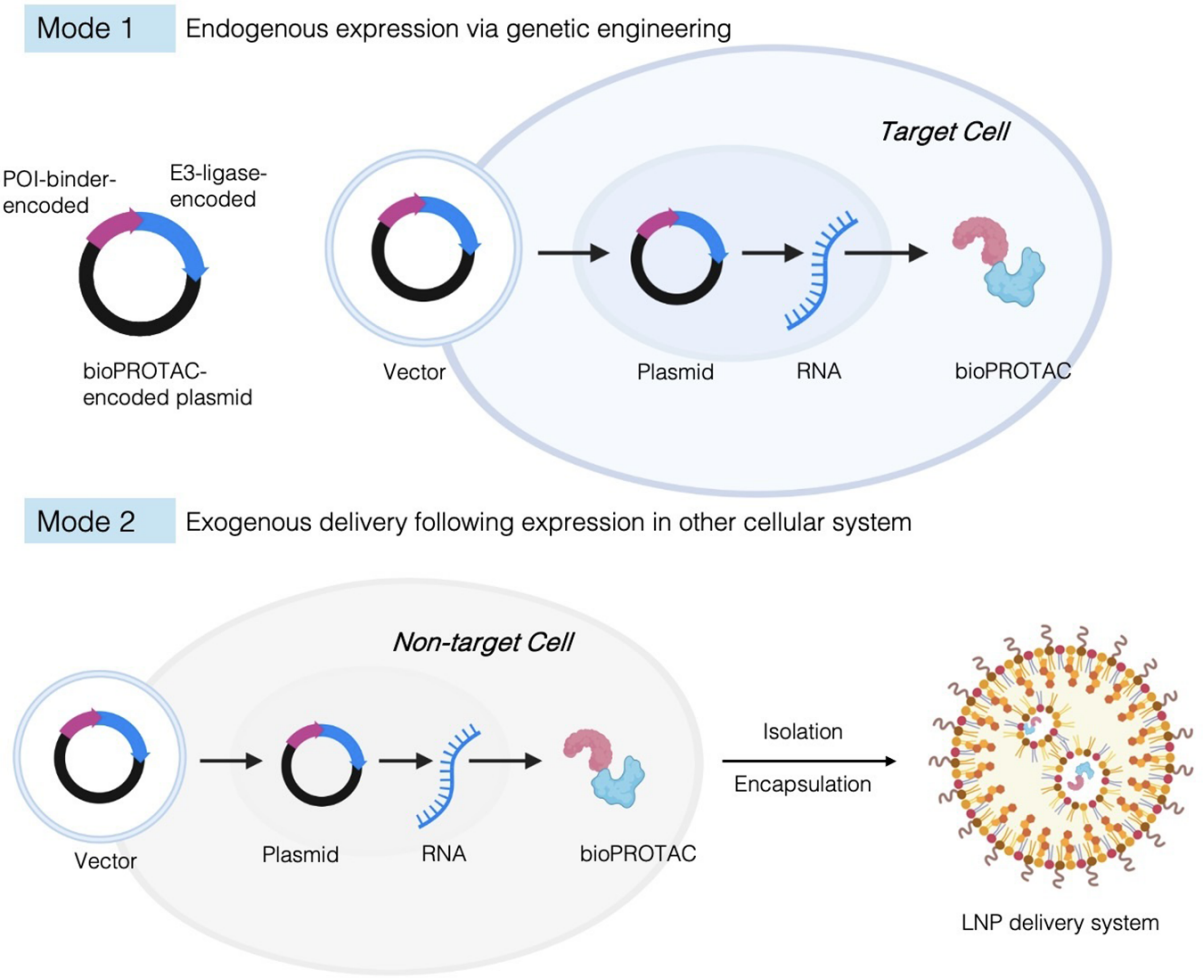
Design and delivery strategies of the bioPROTACs. Schematic illustration of protein-based PROTACs (bioPROTACs), in which amino acid-encoded POI binders and E3 ligase recruiters are fused into protein-like degraders. Mode 1: Endogenous expression via genetic engineering. Mode 2: Exogenous delivery following expression in nontarget cellular systems. Created in BioRender. LU, K. (2026) https://BioRender.com/s8169vv

Fletcher et al. developed a TRIM21-based bioPROTAC to target the RNA-binding protein HuR, a ubiquitously expressed regulator whose overexpression is associated with aggressive tumors and poor prognosis.^80^ A high-affinity single-domain antibody (VHH) against HuR was identified and shown to disrupt HuR–RNA interactions. Fusion of this VHH to TRIM21 enabled the efficient degradation of endogenous HuR, leading to broad remodeling of the HuR-regulated proteome. Importantly, HuR degradation reversed tumor-promoting phenotypes in vivo, demonstrating a clear therapeutic benefit. This work highlights bioPROTACs as a modular and powerful strategy to degrade otherwise intractable targets, such as HuR.

Phase separation is the creation of two distinct phases from a single homogeneous mixture. It drives the formation of biomacromolecular condensates that function as compartments for the selective enrichment of specific molecules.^81^ This principle has been harnessed to increase local molecular concentration, and to improve the efficiency of biological reactions.^82^ Interestingly, Yu et al. introduced an intrinsically disordered region in the PROTAC to replace the original position of the linker.^83^ The DNA coding for the degrader was constructed into a plasmid and delivered through lipid nanoparticles. The droplets formed in the PROTAC-transfected cells partially colocalized with the proteasome, demonstrating the success of co-condensation of POI with the proteasome. Compared to its analogue with the ordinary linker, phase separation showed a more efficient MDM2 degradation.

Delicate controls of POI degradation can be achieved through the logic-gated design of the bioPROTAC function. Yang et al. reported a programmable bioPROTAC platform that uses Sortase A-mediated cleavage and ligation to enable logic-gated control of targeted protein degradation in mammalian cells.^84^ Their “LASER” system allows conditional, switchable degradation outcomes and multitarget modulation by integrating protease inputs with bioPROTAC activity.

Endogenous expression via genetic engineering faces several challenges, including errors in translation and folding, poor mRNA stability, and low expression in mammalian cells.^85,86^ To address these concerns, exogenous delivery following expression in other cellular systems is a direct approach (Figure 6). Chan et al. complexed engineered bioPROTACs with cationic and ionizable lipids via electrostatic interactions for cytosolic delivery and rapid POI degradation. Anionic polypeptides were fused to the PROTAC sequence to facilitate complexing with cationic lipids.^85^

Direct covalent labeling of proteins provides a novel strategy for protein-engineering-based design of biodegraders. Chen et al. engineered platelets by covalently labeling heat shock protein 90 with a POI binder for the degradation of either intracellular or extracellular POIs.^87^ The “living” degrader could home to the postsurgical wound site and achieve in vivo bromodomain-containing protein 4 or PD-L1 degradation for breast cancer treatment.

## 5 Future Perspective

Early reports of pPROTACs were initially constrained by the intrinsically poor druggability of peptides.^1^ However, decades of progress in peptide drug development have generated a rich toolbox to address classical limitations of peptide drugs, including permeability, stability, and systemic exposure.^3^ Especially, the nanoplatform-based delivery strategies have substantially helped alleviate biological barriers, providing a mature and systematic framework for in vivo therapeutic application. pPROTACs are revealing unique opportunities by leveraging peptide specificity, modularity, and genetic encodability to target “undruggable” proteins, mutant variants, and even noncanonical substrates. Their adaptable interaction surfaces and compatibility with conjugation and synthetic biology expand the degradable target landscape and conceptual framework of a TPD.

Currently, the continuous expansion of linker chemistries, E3 ligase recruiters, and CPP motifs has been significantly accelerated by AI, broadening the design flexibility of pPROTACs. These advances have endowed pPROTACs with theoretical targetability toward a wide spectrum of intracellular proteins. Beyond their targeting scope, pPROTACs benefit from exceptional structural modularity and compatibility with diverse bioengineering technologies, which, in turn, define a broad and rapidly expanding research frontier. As a result, the cross-disciplinary integration of pPROTACs with fields such as nanomedicine, synthetic biology, and chemical biology has become increasingly active.

Programming stimulus responsiveness into living cells and biohybrid constructs is galvanizing many advanced bioengineering applications.^88^ Degradation represents a powerful biological impact through the complete blockage of protein function, which would benefit from programmed activation only in response to defined combinations of biochemical cues. The compatibility between pPROTAC and bioengineering enables the development of programmable degradation systems in which peptide degrader activity can be gated by user-defined logic circuits or triggered conditionally by specific biological inputs such as disease-associated enzymes, metabolites, or microenvironmental cues. Looking forward, the integration of logic-gated control and environment-responsive elements into pPROTAC design promises spatially and temporally precise protein degradation, expanding therapeutic and diagnostic opportunities beyond constitutive degrader activity.

From another perspective, as a field of pioneering innovations within the TPD paradigm, pPROTAC research has also given rise to several proof-of-concept strategies that depart from the conventional “all-in-one” molecular architecture. In these designs, functional components are not necessarily confined within a single molecular entity, and such modular or distributed systems have demonstrated enhanced degradation efficiency and improved target-site retention, compared with classical pPROTAC constructs. Notably, a next-generation TPD platform, synthetic peptide-programmed lysosome-targeting chimeras (SPYTACs), has been reported by Teng et al., leveraging the peptide motif that binds to low-density lipoprotein receptor-related protein 1 as inducers of both receptor-mediated endocytosis and blood-brain-barrier transcytosis.^90^ SPTACs targeted at cerebral amyloid-β and showcased superior safety profiles. Its high modularity and genetic encodability confer therapeutic versatility and translational potential across diseases, supporting the advantages of peptide-based TPD platforms.

In parallel, bioPROTACs, which exploit full-length E3 ligase complex components rather than minimal peptide motifs, are emerging as a complementary TPD modality. Owing to their broad target scope and simplified design principles, bioPROTACs hold promise as versatile biological tools for reversible and controllable protein knockdown, thereby expanding the functional landscape of TPD technologies.^49,80,85^

Despite these advances, intracellular delivery and in vivo stability remain the primary bottlenecks for the clinical translation of pPROTACs. Accordingly, a deeper integration of pPROTAC molecular design with formulation and delivery sciences will be critical to unlock their therapeutic potential. Looking ahead, a shift from sequence-centric optimization toward systemic design might be critical for the pPROTAC field. Rather than treating delivery and stability as “downstream” hurdles, future development should embed these considerations in the earliest stages of molecular engineering. This includes exploiting self-regulating architectures, environmental-responsive activation, and programmable degradation logic to align the pPROTAC function with biological context. Besides, advances in responsive chemistries and modular delivery platforms are expected to expand the therapeutic potential of pPROTACs.

The progression of pPROTACs toward clinical application will require the establishment of foundational evaluation frameworks that jointly consider PROTAC-specific and peptide-specific attributes. Key parameters include dose–response relationships, degradation kinetics, plasma half-life, biodistribution, and immunogenicity.^10,11,89^ Moreover, early incorporation of translational considerations, such as manufacturability, scalability, and clinically feasible dosing strategies, will be essential to define realistic development pathways from concept studies to viable therapeutic candidates.

Overall, pPROTACs offer unprecedented opportunities to engage biologically intractable targets through programmable and modular design. While substantial challenges remain in delivery, stability, and translational implementation, rapid advances in protein engineering, peptide chemistry, and delivery sciences are steadily reshaping the landscape. The convergence of these disciplines is expected to enable pPROTACs to evolve from experimental tools to versatile therapeutic platforms with tunable specificity and controlled degradation profiles. As design principles mature and enabling technologies continue to advance, pPROTACs are poised to make breakthroughs in the next generation of precision medicines.
